# Meeting the home-care needs of disabled older persons living in the community: does integrated services delivery make a difference?

**DOI:** 10.1186/1471-2318-11-67

**Published:** 2011-10-26

**Authors:** Nicole Dubuc, Marie-France Dubois, Michel Raîche, N'Deye Rokhaya Gueye, Réjean Hébert

**Affiliations:** 1 Faculty of Medicine and Health Sciences, Université de Sherbrooke, 3001 12th Avenue North, Sherbrooke, Quebec, J1H 5N4, Canada; 2 Research Centre on Aging, University Institute of Geriatrics of Sherbrooke, 1036 Belvedere South, Sherbrooke, Quebec, J1H 4C4, Canada; 3 Faculty of Arts & Faculty of Sciences, Université de Saint-Boniface, 200, Avenue de la Cathédrale, Winnipeg, MB, R2H 0H7, Canada

## Abstract

**Background:**

The PRISMA Model is an innovative coordination-type integrated-service-delivery (ISD) network designed to manage and better match resources to the complex and evolving needs of elders. The goal of this study was to examine the impact of this ISD network on unmet needs among disabled older persons living in the community.

**Methods:**

Using data from the PRISMA study, we compared unmet needs of elders living in the community in areas with or without an ISD network. Disabilities and unmet needs were assessed with the Functional Autonomy Measurement System (SMAF). We used growth-curve analysis to examine changes in unmet needs over time and the variables associated with initial status and change. Sociodemographic characteristics, level of disability, self-perceived health status, cognitive functioning, level of empowerment, and the hours of care received were investigated as covariates. Lastly, we report the prevalence of needs and unmet needs for 29 activities in both areas at the end of the study.

**Results:**

On average, participants were 83 years old; 62% were women. They had a moderate level of disability and mild cognitive problems. On average, they received 2.07 hours/day (SD = 1.08) of disability-related care, mostly provided by family. The findings from growth-curve analysis suggest that elders living in the area where ISD was implemented and those with higher levels of disability experience better fulfillment of their needs over time. Besides the area, being a woman, living alone, having a higher level of disability, more cognitive impairments, and a lower level of empowerment were linked to initial unmet needs (r^2 ^= 0.25; p < 0.001). At the end of the study, 35% (95% CI: 31% to 40%) of elders with needs living in the ISD area had at least one unmet need, compared to 67% (95% CI: 62% to 71%) in the other area. In general, unmet needs were highest for bathing, grooming, urinary incontinence, walking outside, seeing, hearing, preparing meals, and taking medications.

**Conclusions:**

In spite of more than 30 years of home-care services in the province of Quebec, disabled older adults living in the community still have unmet needs. ISD networks such as the PRISMA Model, however, appear to offer an effective response to the long-term-care needs of the elderly.

## Background

In Canada, home-care expenditures represent less than 5% of total health-care spending [1]. Based on data from the 2003 Canadian Community Health Survey (CCHS), Carrière [2] revealed that 33% to 67% of seniors with activities of daily living (ADL) or instrumental activities of daily living (IADL) needs did not receive any form of home care in preceding year. Using the same data, Busque [3] reported that, among elderly who needed assistance, 17.7% had at least one unmet need. A prevalence of 9% for ADL/IADL needs was found by Forbes et al. [4] in a subsample of Canadians diagnosed with dementia. An unmet need is generally defined as a person needing help but reporting not receiving any or enough help [3,5]. Basic ADLs comprise everyday living tasks and are commonly represented by the Katz's index, which includes feeding, bathing, dressing, toileting, continence, and transferring [6]. IADL refers to the periodic routine activities and usually cover the eight activities described by Lawton and Brody: meal preparation, shopping, housekeeping, laundering, using the phone, managing medications, managing money, and using transportation [7]. Activities like walking inside or outside are also considered in some scales. In a study conducted in the province of Quebec, Canada, Lèvesque et al. [8] reported an overall prevalence of perceived unmet ADL/IADL needs of 26% in a sample of people aged 75 years or over. These results are consistent with those obtained in other countries where many community-dwelling older persons live without the ADL/IADL assistance they need. Unmet needs could be reported for any ADL or IADL, and the prevalence of *at least one *unmet ADL or IADL need has been estimated at between one-fifth and one-half [9-22], depending on the sample characteristics, the definition of unmet needs, and which ADL/IADL are considered.

A number of studies have found that rates of unmet needs increased as the number of ADL/IADL limitations increased [9,10,14,16,17,21]. The number of chronic health conditions, gender, ethnicity, marital status or living alone, family income, and limited insurance coverage were also associated with an increased likelihood of unmet needs [16-18,21-23]. Unmet needs related to ADLs and IADLs are not without consequences for frail elders and the health-care system. For example, unmet ADL needs were associated with many negative health-related events such as having pressure ulcers and contractures, losing weight, falling, incontinence, depression, and death [9-11,13,14,24]. As for the health-care system, unmet ADL needs were also associated with increases in the number of physician visits, emergency-department visits, nursing-home placement, and hospitalizations [9,11,14,19]. Thus, these negatives consequences may inhibit benefits that we can expect from the home-care sector [25].

During the last decade in Canada, while the average health spending increased by an average of 2.2% annually, home-care delivery grew by at an annual rate of about 9% [26]. So far, this increased investment does not seem to have improved the situation with regards to needs of disabled older persons living in the community. Home-care programs provide a wide range of care and services (medical to social), which involve numerous care providers and partners. Because of the way in which the services are organized, fragmentation of care is often identified as a recurrent problem that might result from this situation [27]. To resolve fragmentation problems, many projects for integrated-service-delivery (ISD) networks were implemented in the last decade. Although these projects vary according to eligibility criteria, types of integration (linkage, coordination, or full integration), and financing mechanisms, they have highlighted many positive impacts such as improvement in elder satisfaction [28-31], improvement in elder empowerment [30], reduction of caregiver burden [32], decrease in functional decline [30,32,33], reduction in hospital use [28-30,33,34], reduction in emergency-department visits [30], reduction in nursing home utilization [28,29,32-34], and reduction in costs [29].

Surprisingly, even though all these programs aimed at reducing the fragmentation of care, consolidating the range of services, and promoting a better match of resources to the older person's needs, only the Wisconsin Partnership Program (WPP) (a variation of the PACE Program), [12], has investigated unmet needs for ADL/IADL disabilities as a main outcome of an ISD network, but failed to achieve an impact. The use of a cross-sectional design, short exposure time (just over a year), and inadequate physician participation could explain the lack of differences. Two other studies addressed unmet needs, although it was not their primary interest. Sands et al. [19] showed an interesting decline in the use of acute care after six weeks of receiving PACE services for those experiencing ADL unmet needs before enrolment. Yet this study was limited to five ADLs, did not use a comparison group, and retrieved information from the participants' medical records. In another study conducted with 800 members of four Social HMOs, Leutz and Capitman [35] found that, among those who wanted help from SHMOs, many did not get any or enough help in all eleven areas, especially for nonmedical transportation (58%), managing money (51%), feeling lonely (60%), and emotional support (62%).

Our study aimed at increasing our understanding of the role of integrated models of services in meeting the home-care needs of disabled older persons. This paper uses data from the Program of Research to Integrate Services for the Maintenance of Autonomy (PRISMA) study, conducted between 2001 and 2006 [30]. It first reports the impact of the PRISMA Model-an integrated-service-delivery (ISD) network-on unmet needs among older adults living in the community. Second, it identifies the correlates of initial status and change in unmet needs. Lastly, it examines the prevalence of unmet needs separately for 29 activities at the end of the study. The PRISMA Model provides data from the ISD network most recently implemented in Canada. Different from fully integrated models, which work in parallel with their usual health systems, the PRISMA Model is a coordination-type model of integration in which the ISD network was embedded within the health- and social-care system using all the public, private, and voluntary health- and-social-services organizations involved in caring for older people in a given area [30]. The mechanisms and tools developed and implemented by PRISMA are: 1) coordination between decision-makers and managers at the regional and local levels, 2) use of a single entry point, 3) a case-management process, 4) individualized service plans, 5) a single assessment instrument coupled with a management system based on client disabilities, and 6) a computerized clinical chart allowing communication between institutions and clinicians for client monitoring purposes [30,36].

It should be noted that, in the province of Quebec, Canada, health- and social-services centers (HSSCs) are responsible for delivering home-care services at the local level. In 2005, HSSCs were created out of the merger of local community services centers, residential and long-term-care centers, and, in most cases, a hospital [37]. After an individual clinical assessment of a person's biopsychosocial needs has been performed, care and services are provided by health professionals, including physicians, nurses, social workers, physiotherapists, occupational therapists, nutritionists, and homemakers. In addition, given available resources, HSSCs provide, in collaboration with their partners (community groups, the social-economy sector, the private sector), a wide range of care and services, including personal and domestic assistance, specialized care and services, civic-support services, accompaniment on outings, friendly visits, and respite [38].

## Methods

### Design and settings

The PRISMA study used a quasi-experimental design (pretest, multiple posttests with a control group) to investigate the effectiveness of the PRISMA model of integration. The PRISMA ISD network was implemented in three zones (urban, semi-urban, and rural) in one area of the province of Quebec (experimental area). Three control zones were selected in another area of Quebec, based on their similarities with the experimental zones in terms of demographic variables and health-services availability. Study areas were broadly representative of the variety of services offered provincially by both the public and private sectors. During the study period, the implementation rate, as measured by formal indicators [39], increased from 22% at the beginning of the study to 67% after one year, 73% after two years, and 77% after three years [36].

### Participants and data-collection procedures

Details of the sampling procedures have been described elsewhere [30,40]. Briefly, samples were selected in each area and they were restricted to adults aged 75 or over, living in the community, at risk of functional decline and able to speak and understand French. A total of 920 persons were recruited throughout 2001 (first annual wave) and followed yearly for four years. During 2003 and early 2004 (third annual wave), 581 additional participants were recruited and monitored for two years (three waves), the last ones being measured at the onset of 2006. Study participants were interviewed in their own settings at baseline and yearly for the duration of their participation. They were also contacted by phone every two months to collect their use of health and social services. Proxy interviews with the person who provided the most assistance to the subject were conducted for those who were unable to answer. The Ethics Review Board of the Health and Social Services Centre - University Institute of Geriatrics of Sherbrooke (HSSC-UIGS) approved all study procedures and all participants signed a consent form.

### Measurements

Trained research assistants used uniform, standardized, and well-validated instruments. We present here only measurements relevant to this paper. See Hèbert et al [40], for full details on all instruments used in the PRISMA study. Besides the principal independent variable (living in an area with or without an ISD network) and the outcome variable of unmet needs, the sociodemographic variables considered in this analysis included age, gender, marital status, level of education, type of housing (home or collective dwelling), living arrangements (alone or not), the availability of an informal caregiver, and living in an urban, semi-urban, or rural environment. Other variables such as level of functional disability, self-perceived health status, cognitive status, level of empowerment, and the time of care provided by the public and private sectors as well as by the family were considered.

As specified by Williams, Lyons & Rowland [5], when studying unmet needs, it is crucial to understand how disability is measured, and how unmet needs related to disability are defined and measured. The levels of functional disability and unmet needs of PRISMA participants were evaluated with the SMAF (French acronym for Functional Autonomy Measurement System). The SMAF does not cover only ADLs and IADLs, but represents a multidimensional needs assessment covering five domains of activity that were proposed in the WHO classification of disabilities during its development [41,42]. The five domains comprise 29 items covering ADLs (eating, washing, dressing, grooming, urinary function, bowel function, toileting), mobility (transferring, walking inside, installing a prosthesis or an orthosis, propelling a wheelchair inside, using the stairs, getting around outside), communication (seeing, hearing, speaking), mental functions (memory, orientation, comprehension, judgment, behavior), and IADLs (housekeeping, meal preparation, shopping, laundry, telephone, transportation, managing medication, budgeting). For each function, the disability is scored from 0 (independent) to 3 (dependent) by a professional according to precise criteria derived from information obtained through interviews with and observation of the participant or by interviewing a third party. Note that items are framed to capture disabilities related to interactions between individuals and their environments, which, in turn, consist of complicated arrays of social and cultural components. A total score between 0 and 87 is obtained by summing all items, with higher scores representing decreased functional ability.

Available resources to compensate for each disability can also be evaluated while completing the SMAF and a score representing *unmet needs *is deducted. If available resources compensate or if no disability is measured for a given function, the score for that item is zero and we consider that the needs have been fulfilled. If not, the score is equal to the disability score. This way of proceeding investigates unmet needs as both the absence of and insufficient assistance with any SMAF item. An unmet need score ranging from 0 to 87 is then obtained. Assessors started completing this part of the SMAF in 2002 (second annual waves of the PRISMA study), so that four annual waves of data are available for this variable.

For each disability item, the type of available resource (public sector, private sector, or family) and the frequency of help were also gathered. They were entered into regression equations to estimate the hours of care per day provided by each resource. These regression equations were developed with data from a previous study in which 1997 subjects were assessed independently with the SMAF and the Modified CTMSP [43], an instrument that uses a two-step procedure to determine the resources required and received by each subject. First, a nurse used a standardized form to assess the individual's social, medical, and psychological needs, and collected information on the resources actually received by the subject. Second, these data were reviewed and analyzed by a team composed of another nurse and a social worker. Using a standardized procedure, the team determined the hours of care required and received. The test-retest and inter-rater reliability intraclass correlation coefficients of the data collection procedure were established at 0.91 and 0.95, respectively. The intra-team and inter-team reliability was also very good, with coefficients of 0.91 and 0.92, respectively [44]. Using the disability score on SMAF items, the SMAF explained 51% of the variance in skilled nursing-care time (p < 0.006) and 87% of the variance in unskilled care (personal care and instrumental tasks) (p < 0.005) [45]. The SMAF instrument has been the subject of many validity and reliability studies over the past 20 years [42,46,47]. The intraclass correlation coefficient (ICC) was estimated to be 0.95 for test-retest reliability and 0.96 for interrater reliability [47]. The concomitant construct validity was confirmed with the correlation between the SMAF and Functional Independence Measure [48] (r = 0.94) and Barthel Index [49] (r = 0.92). The responsiveness of the scale has been studied and the Guyatt index was 14.53 [46].

The self-perceived health status was assessed by asking participants "Compared to persons of your age, in general, how do you rate your own health?" The response options were "excellent, good, fair, or poor". Cognitive status was assessed with the Mini-Mental State Examination (MMSE) [50]. This short test comprises 11 items assessing orientation about temporal and spatial information, attention, immediate and short-term recall, language, and the ability to follow simple verbal and written commands. The score ranges from 0 (worst) to 30 (best).

Lastly, the level of empowerment was determined with the Health Care Empowerment Questionnaire (HCEQ), which consists of 10 statements, each answered on two four-grade scales: one for perception; the other for importance [51]. The combination of these two scales gives a score for each statement varying from 1 (worst) to 16 (best), and the total score is the mean over all statements. The HCEQ covers three dimensions: the patient's involvement in the decisional process, the patient's involvement in interactions with professionals, and the patient's degree of control in regard to care and services received.

### Statistical analysis

Descriptive statistics (means and standard deviations or percentages) were used to summarize data for participants living in areas with and without an ISD network. Areas were compared using Student's *t *test for continuous variables or chi-square for categorical variables. The Chi-square test or Fisher's exact test were also used to compare areas with regards to the percentage of needs and unmet needs (scores > 0) in five domains (ADLs, mobility, communication, mental functions, and IADLs) and for each SMAF item.

We used growth-curve analyses to examine the change in unmet needs over time and the variables associated with initial status and change [52-54]. Growth models, also called multilevel models for change, took into account all available measures of participants with incomplete follow-up. They addressed within-person and between-person questions about change simultaneously with a pair of submodels. The level-1 submodel describes how unmet needs changed over time for each person. The level-2 submodel allows for studying the effect of an ISD network on this change. Other potential correlates of individual change were also included in level 2. Time was scaled as the number of days since January 1, 2002, allowing the number and spacing of measurement occasions to vary from one subject to another [54]. Since the dependent variable was not normally distributed, it was log-transformed. We first modeled a quadratic relationship of unmet needs with time, but, since the quadratic term was not significant at the 5% level, a linear model was chosen for parsimony. The analysis had four main steps, and parameters were estimated with the method of maximum likelihood using SAS PROC MIXED, version 9.1 (SAS Institute Inc, Cary, NC).

In the first step, we used an unconditional means model to partition and quantify the total variability in unmet needs into its within- and between-persons components, without regard to time. We used the following two equations:

$$\begin{array}{c} {\text{Y}}_{ij}=p_{0i}+\varepsilon_{ij}\;\text{at}\;\text{level}\;\;1,\\ \text{and}\;\;p_{0i}=\gamma_{00}+\zeta_{0i}\;\text{at}\;\text{level}\;\;2,\\ \text{with}\;\varepsilon_{ij}\sim \;\text{N}\left(0,{\sigma^{2}}_{\varepsilon }\right)\;\text{and}\;\;\zeta_{0i,}\sim \text{N}\left(0,{\sigma^{2}}_{0}\right) \end{array}$$

where Y*_ij _*= log (unmet needs score*_ij _*+ 1) for subject *i *, (*i *= 1, ·, *n*) at time *j *(*j *= 1, ·, t*_i _*which represents the number of measurement occasions for subject *i*), π_0*i *_is the mean for subject *i*, γ_00 _is the grand mean in the population, σ^2^_0 _represents the within-person variance, and σ^2^_0 _represents the between-person variance. These variances were used to estimate the intraclass correlation coefficient *ρ*, which describes the proportion of the total outcome variation that lies between people.

$$\rho =\frac{\sigma_{0}^{2}}{\sigma_{0}^{2}+\sigma_{\varepsilon }^{2}}.$$

Next, in step 2, we used an unconditional growth model to introduce the TIME predictor (T*_ij _*= number of days since January 1, 2002, for subject *i *at measurement time *j*) into the level-1 submodel. This model was defined by a set of three equations:

$$\begin{array}{c} {\text{Y}}_{ij}=\pi_{0i}+p_{1i}{\text{T}}_{ij}+\varepsilon_{ij}\;\text{at}\;\text{level}\;1,\\ p_{0i}=\gamma_{00}+\zeta_{0i}\;\text{and}\;p_{1i}=\gamma_{10}+\zeta_{1i}\;\text{at}\;\text{level}\;2,\\ \text{with}\;\varepsilon_{ij}\sim \text{N}\left(0,{\sigma^{2}}_{\varepsilon }\right)\;\text{and}\;\left[\begin{array}{c} \zeta_{0i}\\ \zeta_{1i} \end{array}\right]\sim N\left(\left(\begin{array}{c} 0\\ 0 \end{array}\right),\left[\begin{array}{c} {\sigma^{2}}_{0}\;\sigma_{01}\\ \sigma_{01}\;{\sigma^{2}}_{1} \end{array}\right]\right) \end{array}$$

The slopes π_1*i *_for time, defined as random, reflect the rate of change over time in the log-transformed unmet need score for each subject *i*, while γ_00 _and γ_10 _represent, respectively, the average intercept and average slope over all subjects. The level-2 variance components σ^2^_0 _and σ^2^_1 _represent the between-person variability in initial status and rates of change, respectively.

In step 3, we conditioned the model with the AREA*_i _*variable (subject *i *living in an area with or without an ISD network) as a predictor of both initial status and rate of change in log-transformed unmet need scores. Because the AREA*_i _*variable was constant over time for each subject, it was entered at level 2. The equations were:

$$\begin{array}{c} {\text{Y}}_{ij}=\pi_{0i}+\pi_{1i}{\text{T}}_{ij}+\varepsilon_{ij}\;\text{at}\;\text{level}\;1,\\ \pi_{0i}=\gamma_{00}+\gamma_{01}\text{ARE}{\text{A}}_{i}+\zeta_{0i}\;\text{and}\;\pi_{1i}=\\ \gamma_{10}+\gamma_{11}\text{ARE}{\text{A}}_{i}+\zeta_{1i}\;\text{at}\;\text{level}\;2,\\ \text{with}\;\varepsilon_{ij}\sim \text{N}\left(0,{\sigma^{2}}_{\varepsilon }\right)\;\text{and}\;\left[\begin{array}{c} \zeta_{0i}\\ \zeta_{1i} \end{array}\right]\sim N\left(\left(\begin{array}{c} 0\\ 0 \end{array}\right),\left[\begin{array}{cc} {\sigma^{2}}_{0} & \sigma_{01}\\ \sigma_{01} & {\sigma^{2}}_{1} \end{array}\right]\right) \end{array}$$

Parameters γ_01 _and γ_11 _in the level-2 submodels represent the effects of AREA*_i _*on initial status and individual change trajectories. The level-2 variance components represent the between-person variation in change trajectories that remained unexplained by the level-2 predictor AREA*_i_*.

Finally, in step 4, we evaluated the fixed effect of other potential correlates on initial status or rate of change in log-transformed unmet need scores. All variables were first tested one at a time and those with a significant effect on either initial status or rate of change were kept for this fourth step. Continuous variables were centered on their mean value. Each variable that was constant over time (noted Z*_li _*= *l*^th ^variable (l = 1, ·, *q*) for subject *i*) was introduced in level 2. Time-dependent variables (noted X*_kij _*= *k*^th ^time-varying variable (*k *= 1, ·, *p*) for subject *i *at measurement time *j*) were introduced in level 1, giving the following set of equations:

$$\begin{array}{c} {\text{Y}}_{ij}=p_{0i}+p_{1i}{\text{T}}_{ij}+\left[\gamma_{2}{\text{X}}_{1ij}+\cdot \cdot \cdot +\gamma_{\left(\text{p}+1\right)}{\text{X}}_{pij}\right]+\\ \;\left[\gamma_{\left(\text{p}+2\right)}{\text{X}}_{1ij}+\cdot \cdot \cdot +\gamma_{\left(2\text{p}+1\right)}{\text{X}}_{pij}\right]*{\text{T}}_{ij}+\varepsilon_{ij}\\ \pi_{0i}=\gamma_{00}+\gamma_{01}\text{ARE}{\text{A}}_{i}+\gamma_{02}{\text{Z}}_{1i}+\cdot \cdot \cdot +\gamma_{0\left(\text{q}+1\right)}{\text{Z}}_{qi}+\zeta_{0i}\\ \pi_{1i}=\gamma_{10}+\gamma_{11}\text{ARE}{\text{A}}_{i}+\gamma_{12}{\text{Z}}_{1i}+\cdot \cdot \cdot +\gamma_{1\left(\text{q}+1\right)}{\text{Z}}_{qi}+\zeta_{1i}\\ \text{with}\;\varepsilon_{ij}\sim \text{N}\left(0,{\sigma^{2}}_{\varepsilon }\right)\;\text{and}\;\left[\begin{array}{c} \zeta_{0i}\\ \zeta_{1i} \end{array}\right]\sim N\left(\left(\begin{array}{c} 0\\ 0 \end{array}\right),\left[\begin{array}{c} {\sigma^{2}}_{0}\;\sigma_{01}\\ \sigma_{01}\;{\sigma^{2}}_{1} \end{array}\right]\right) \end{array}$$

This model was reduced by backward elimination for parsimony; the relative fit of two nested models was compared by computing the difference in their deviance statistics. This difference follows a chi-square (*χ*^2^) distribution with *df *equal to the difference in the number of parameters in both models. The significance level was set at 5%. In each step, pseudo-R^2 ^statistics were computed to quantify the explained variability [54].

## Results

### Participant characteristics

Since unmet needs were assessed from the second wave of the PRISMA study, a total of 746 persons (419 with an ISD network and 327 without an ISD network) with at least one measure were available for analyses. The characteristics of participants in the second wave from both areas are detailed in Table 1. On average, participants were 83 years, two-thirds were women, 46% were married, 42% were living in an urban area, 31% were living alone, the level of education was 7 years, and most of the participants had an informal caregiver (90%). They had moderate level of disabilities, mild cognitive problems, and a moderate level of empowerment. Most of them (63%) perceived their health status as good or excellent compared to other people of their age. Regarding the time of care, participants received an average of 2.07 hours/day (SD = 1.08) of care, assistance, and support related to disabilities. The major portion (54% and 63% for areas with and without an ISD network, respectively) was provided by the family, followed by the private sector (41% and 32% for areas with and without an ISD network, respectively). Only 5% of care in both areas was supplied by the public sector. Lastly, though there were no statistically significant differences between areas for most variables, the participants in the area with an ISD network were slightly but significantly older, and had better cognitive functions than participants from the area without an ISD network. These differences, however, do not appear to be clinically significant. Significantly more participants from the ISD network were living in a collective dwelling compared to an individual home.

**Table 1:** Comparison of sociodemographic, clinical characteristics, time for care and services, and needs between participants

AREA	Area With ISD* (n = 419)	Area Without ISD (n = 327)	p value
**Sociodemographic characteristics ◇**			

Age on January1, 2002	83.00 (4.71) §	82.16 (4.78)	0.018
Gender (Female)	271 (64.7%)	195 (59.6%)	0.158
Marital status (Widowed)	209 (50.0%)	151 (46.2%)	0.300
Living alone	137 (32.8%)	94 (28.8%)	0.238
Years of education	6.56 (2.98)	6.76 (3.30)	0.388
Living in collective dwelling	153 (36.5%)	80 (24.5%)	< 0.001
Living Area			
- Urban	171 (40.8%)	139 (42.5%)	
- Semi-urban	114(27.2%)	93 (28.2%)	
- Rural	134(32.0%)	95 (29.1%)	0.690
Having an informal caregiver	376 (89.7%)	297 (90.8%)	0.619

**Clinical characteristics**			

Disability (SMAF)	18.65 (11.35)	20.04 (12.94)	0.124
Cognitive functioning (MMSE)	25.32 (4.42)	23.96 (6.88)	0.002
Excellent or good health status †	256 (61.5%)	212 (65.2%)	0.301
Empowerment (HCEQ)	7.25 (2.26)	7.05 (2.38)	0.242

**Time of care and services**			

Total time received (hours/day)	2.07 (1.03)	2.07 (1.15)	0.963
Time provided by the public sector (hours/day)	0.10 (0.30)	0.11 (0.32)	0.810
Time provided by the private sector (hours/day)	0.85 (0.94)	0.65 (0.83)	0.002
Time provided by the family (hours/day)	1.11 (0.88)	1.31 (1.10)	0.009

**Needs and unmet needs**			

% with needs	418 (99.8%)	324 (99.1%)	0.208
% with unmet needs Unmet-needs score	283 (67.5%) 2.12 (2.48)	195 (59.6%) 1.89 (2.54)	0.025 0.02 Δ

* ISD: integrated-service-delivery (ISD) network

◊ measured at wave two of the PRISMA study

§ Mean (SD) for continuous variables; n (%) for categorical variables.

†Subjective health status compared to others of the same age.

p value when comparing the two areas, using Student's *t *test for continuous variables or the Chi square test for categorical variables.

Δ comparing log-transformed scores

### Change in unmet needs over time

**Figure 1:**
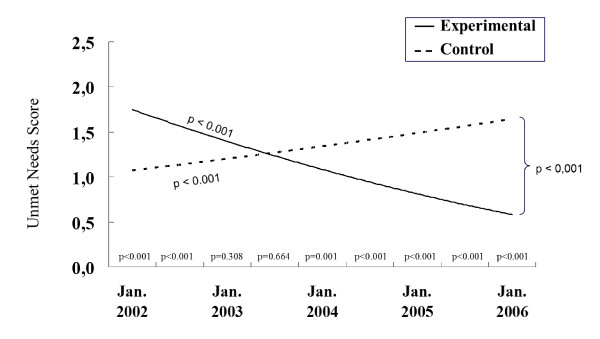
**Evolution of the unmet needs score according to area with or without ISD network**.

Step 1 of our growth-curve analysis revealed that the average unmet needs score was statistically different from zero, that it varied within persons (thus over time), and that subjects differed from each other in these variations. The estimate of the intraclass correlation coefficient is 0.46, indicating that 46% of the total variation in the dependent variable is attributable to differences between subjects. In step 2, we introduced TIME into the model. A statistically significant negative coefficient for TIME (p < 0.001) indicated that, on average, unmet needs decreased over time. In other words, needs were more fulfilled over time. Linear time explains 1.2% of the total variability and 7% of the within-person variation in the dependent variable. In step 3, we added the AREA variable as a predictor of both initial status and rate of change in the log-transformed unmet-need scores. A negative coefficient for AREA on initial status indicated that, on average, people living in the control area initially had fewer unmet needs than those living in the experimental area. Yet the coefficient for AREA is positive for rate of change (p < 0.001), meaning the decrease over time was smaller in the control area. AREA explains 5% of the variation in initial status and 65% of the variation in rates of change. Figure 1 illustrates the mean change in unmet needs over time for each area. Step four was related to the addition of covariates. Concerning variables that were first tested one at a time, level of education was not related to initial status or change, and thus was not introduced into the model. Because the variables "living alone" and "type of housing" were highly correlated, we chose to retain only "living alone." Lastly, although significantly related to either initial status or change when considered separately, age, marital status, living in an urban or rural environment, self-perceived health status, and hours of formal or informal services were not significant in the final model shown in Table 2. For variables associated with initial status, a positive coefficient means that the variable is associated with a higher likelihood of having unmet needs. Therefore, being a woman, living alone and in the experimental region, and having a higher level of disability (all ps < 0.001) are associated with having initially more unmet needs. Inversely, the negative coefficients indicate that unmet needs were initially higher for people with a lower level of empowerment (p < 0.001) and a lower MMSE score (p = 0.008), meaning more cognitive impairments. Area (p < 0.001) and disability (p = 0.002) were statistically related to change with TIME, suggesting that those living in the experimental area and with higher levels of disability experienced steeper decline of their unmet needs (i.e. better fulfillment of their needs) over time. This model fits better than the model with AREA only, as evidenced by the large drop in deviance statistics when comparing the two models (p < 0.0001).

**Table 2:** Results of the final multilevel model for change in log (1 + unmet needs score)

Fixed Effects		Parameter	Parameter Estimate	P value
Initial status				
	Intercept	γ_00_	0.9526	< 0.001
	Control area	γ_01_	-0.2989	< 0.001
	Disability^§^	γ_02_	0.0305	< 0.001
	Gender (Female)	γ_03_	0.1662	< 0.001
	Living alone	γ_04_	0.1454	< 0.001
	Cognitive functioning^§^	γ_05_	-0.0059	0.008
	Empowerment^§^	γ_06_	-0.0305	< 0.001

Rate of change				
	Intercept	γ_10_	-0.1815	< 0.001
	Control area	γ_11_	0.1815	< 0.001
	Disability^§^	γ_12_	-0.0026	0.002

Variance components				

Level 1:	Within-person	σ^2^_ε_	0.2657	< 0.001
Level 2:	In initial status	σ^2^_0_	0.1767	< 0.001
	In rate of change	σ^2^_1_	0.0069	0.043
	Covariance	σ_01_	-0.0142	0.117

Goodness-of-fit				

	Deviance		6789.3	

Pseudo R^2 ^Statistics				
	R^2^(*y*. %)		25.4%	

^§ ^Centered at their mean value

### Prevalence of unmet needs at the end of the study

Lastly, Table 3 presents the proportion of participants with at least one need (SMAF score > 0) and among those, the proportion with at least one unmet need (score > 0) for both areas at the end of the PRISMA study for each SMAF domain and item. In both areas, all participants had at least one need related to their functional abilities. In the ISD experimental area, most of the participant needs were for ADLs (90%) and IADLs (99%). For other domains, the prevalence ranged from 65% in communication to 70% for mobility and mental functions. For the participants from the area without an ISD network, there were significant needs in ADLs (96%) and IADLs (99%), but also in mobility (95%), while the prevalence of needs in communication (77%) and in mental functions (74%) was lower. Statistically significant differences between areas appear in the ADL, mobility, and communication domains, in which participants living in the area without an ISD network had more needs at the end of the PRISMA study.

**Table 3:** Prevalence of needs and unmet needs for SMAF items (last wave of the study)

SMAF Items	Area With ISD†(n = 395)		Area Without ISD (n = 433)	
	**At Least One Need** **n (%)**	**At Least One** **Unmet Need** **n (%)^§^**	**At Least One Need** **n (%)**	**At Least One** **Unmet Need** **n (%)^§^**

**Total SMAF unmet needs score**	**393 (99.5%)**	**139 (35.5%)**	**434 (100%)**	289 (66.7%)***

**A. ADLs**	**354 (89.6%)**	**111 (31.4%)**	418 (96.3%)***	196 (47.0%)***

Eating	69 (17.5%)	0 (0%)	145 (33.4%)***	8 (5.6%)**
Washing	188 (47.6%)	36 (19.3%)	307 (70.7%)***	75 (24.5%)***
Dressing	133 (33.7%)	0 (0%)	259 (59.7%)***	8 (3.1%)**
Grooming	288 (72.9%)	82 (28.6%)	383 (88.2%)***	128 (33.5%)**
Urinary continence	190 (48.1%)	4 (2.1%)	228 (52.5%)	10 (4.4%)
Fecal continence	158 (40.0%)	2 (1.3%)	205 (47.2%)*	40 (19.5%)***
Using toilet	59 (14.9%)	1 (1.7%)	225 (51.8%)***	7 (3.1%)

**B. Mobility**	**276 (69.9%)**	**8 (2.9%)**	411 (94.7%)***	70 (17.1%)***

Transfers	81 (20.5%)	0 (0%)	308 (71.0%)***	8 (2.6%)**
Walking inside	152 (38.5%)	3 (2.0%)	244 (56.2%)***	14 (5.8%)*
Walking outside	207 (52.4%)	4 (1.9%)	380 (87.6%)***	41 (10.8%)***
Putting on prosthesis or orthosis	11 (2.8%)	0 (0%)	2 (0.5%)**	0 (0%)
Moving around in a wheelchair	35 (8.9%)	3 (8.8%)	56 (12.9%)	1 (1.8%)
Using the stairs	230 (58.2%)	0 (0%)	368 (84.8%)***	23 (6.3%)***

**C. Communication**	**255 (64.6%)**	**28 (11.0%)**	336 (77.4%)***	152 (45.4%)***

Seeing	154 (39.0%)	12 (7.8%)	227 (52.3%)***	77 (34.1%)***
Hearing	188 (47.6%)	17 (9.0%)	249 (57.4%)**	114 (45.8%)***
Speaking	11 (2.8%)	1 (9.1%)	30 (6.9%)**	3 (10.0%)

**D. Mental functions**	**272 (68.9%)**	**3 (1.1%)**	**320 (73.7%)**	25 (7.8%)***

Memory	251 (63.5%)	2 (0.8%)	302 (69.6%)	14 (4.6%)**
Orientation	137 (34.7%)	1 (0.7%)	125 (28.8%)	8 (6.5%)*
Understanding	133 (33.7%)	2 (1.5%)	92 (21.2%)***	13 (14.1%)**
Judgement	133 (33.7%)	1 (0.8%)	111 (25.6%)*	6 (5.4%)
Behaviour	74 (18.7%)	0 (0%)	68 (15.7%)	10 (14.7%)**

**E. IADLs**	**391 (99.0%)**	**20 (5.1%)**	**429 (98.9%)**	52 (12.2%)***

Cleaning the house	357 (90.4%)	4 (1.1%)	404 (93.1%)	8 (2.0%)
Preparing meals	302 (76.5%)	4 (1.3%)	350 (80.7%)	19 (5.4%)**
Shopping	311 (78.7%)	0 (0%)	390 (89.9%)***	1 (0.3%)
Doing the laundry	254 (64.3%)	0 (0%)	309 (71.2%)*	3 (1.0%)
Using the telephone	183 (46.3%)	5 (2.8%)	242 (55.8%)**	6 (2.5%)
Using public transportation	275 (69.6%)	0 (0%)	335 (77.2%)*	6 (1.8%)*
Taking medications	271 (68.6%)	7 (2.6%)	314(72.4%)	20 (6.4%)*
Managing the budget	213 (53.9%)	0 (0%)	258 (59.5%)	2 (0.8%)

† ISD: integrated-service-delivery (ISD) network

^§ ^Among subjects with at least one need

* p < 0.05; ** p < 0.01; *** p < 0.001 when comparing the two areas, using the Chi square or Fisher's exact test.

As regards unmet needs at the end of the study, 35% (95% CI: 31% to 40%) of community-dwelling elders with needs living in the ISD area had at least one unmet need, compared to 67% (95% CI: 62% to 71%) in the other area. In fact, participants from the area without the ISD network had significantly more unmet needs in all five domains of activities. In this area, the highest rates of unmet needs were for hearing (46%), seeing (34%) grooming (34%), bathing (25%), bowel incontinence (20%), walking outside (11%), speaking (10%), and walking inside (6%). Unmet needs were also observed in IADL functions: preparing meals (5%) and taking medications (6%). For the participants living in an area with the ISD network, unmet needs were mostly for grooming (29%), bathing (19%), hearing (9%), speaking (9%), and seeing (8%).

## Discussion

The primary goal of this study was to examine the impact of an innovative model of an ISD network on unmet needs among the elderly living in the community. Using a population-based approach, data from a 3-year follow-up of a randomly selected stratified sample of community-dwelling older persons at risk of functional decline with a longitudinal quasi-experimental design, we found that living in experimental area identified for implementation of an ISD network was associated with initially more unmet needs, but also with a steeper decrease over time.

These findings are probably attributable to a complex relationship between numerous factors. The growth-curve model with AREA only revealed that this factor accounted for 65% of the variation in rates of change in unmet needs. Given that eligibility criteria for home care became more clearly defined over time in the ISD area, frail and disabled elders were perhaps targeted there more rapidly. Furthermore, the significant effect of the PRISMA Model [30] on reducing the prevalence and incidence of functional decline in the experimental group over the last two years might explain part of these results, since the level of disability was consistently reported to be related to unmet needs [9,10,17,21]. In the ISD area, the participants' mean level of empowerment was also higher than in the control area [30]. Consequently, participants in the ISD area were probably more likely to express their needs, to take an active role in obtaining useful information or services, to initiate relevant steps to their health-care situation, or to manage the challenges of their daily life at home. Another impact of the PRISMA Model was that participant satisfaction improved in the ISD area, yet remained unchanged in the control area [30]. Greater satisfaction with services may reflect health care and social services that were better delivered and organized, and could have contributed to reducing unmet needs. Many characteristics of the PRISMA Model, such as the "single entry point," that is, a mechanism for accessing services, the individualized service plan or the model of coordination achieved between public, private, and community services might also have influenced these results. Some interventions such as providing information about available services and how to access them may have supported participants in finding help to better fulfill their needs. In fact, it is difficult to focus on one aspect separately as all six elements of the model interact together. They were also well implemented and all considered important, although improvements are needed to make the individualised service plan more useful and effective in supporting the case manager's work [36,55].

Without more information, it is difficult to point to other factors that might account for the differences in the level of unmet needs between areas. Although we measured many variables related to elder characteristics and some factors linked to the health-care system, the final growth model explained 25% of the variance in unmet needs. This suggests that a broader range of factors needs to be considered. Since we did not address the reasons for unmet needs, we do not know if, or to what extent, professional oversight, service insufficiencies or inaccessibility, other system failure, or personal preferences played a role in unmet needs. In their study of people with dementia, Forbes et al. [4] observed that availability, cost and knowledge of service, and the decision not to seek care were reasons for not receiving care. Nevertheless, potential selection bias was limited, and comparability between areas was enhanced since participants were chosen randomly from the older population, and selection of the control area was based on similarities with the experimental area in terms of demographic, economic, and health-services aspects according a systematic method [30].

The second objective was to identify the correlates of initial status and change in unmet needs. We found that older persons living in the area where the ISD network was expected to be implemented or having higher level of disabilities experienced a steeper decline of their unmet needs over time. Besides the area, being a woman, living alone, having a higher level of disability, greater cognitive impairments, and a lower level of empowerment were linked to initial unmet needs. Identifying related factors and those that could contribute to a greater decrease in unmet needs may provide new perspectives on how to organize home-care services, and, in turn, improve daily life among older persons with disabilities. Factors such as, age, level of education, self-perceived health status, time of care and services received, and type of setting were not significant predictors of either initial status or change in unmet needs. With respect to these variables, some discrepancies were found in previous studies [3,17,18,21,56].

As for other factors identified as correlates in this study, the finding that individuals living alone had more unmet needs is consistent with an extensive body of the literature in the field [10,16-18,21,22]. As reported in two Canadian studies [4,8], being a woman was associated with having more unmet needs. In other studies, various gender-related effects were observed. It must be recognized that, after age 75, women are more likely to be widowed, while older men are more likely to be married and have a spouse who can assist them in the event of disability. Having more cognitive impairments was related to an increase in unmet needs. It seems consistent with the fact that such elders require more care and services than others with a high level of cognitive functioning. It is, however, difficult to make comparisons with others studies, given the differences in methods. First, since many previous studies relied on self-reporting, older persons with cognitive impairment were often excluded [5]. Some studies included people with dementia and used a proxy as respondent. Having a proxy was associated with a reduced likelihood of unmet needs [10,17]. In our study, like in another Canadian study, the presence of a proxy didn't seem sufficient to fulfill all needs for women with dementia [4]. In the other study, although women with dementia reported receiving more services, they also indicated greater unmet home-care needs than men. In fact, we are aware of few studies that have addressed level of cognitive functioning as a predictor of unmet needs. In one study, cognitive function was not associated with unmet needs [18], while van der Roest et al. [57] found that the caregivers of people with severe dementia reported fewer unmet needs for self-care than caregivers of people with mild to moderate dementia. Finally, earlier studies never addressed level of empowerment. It makes sense, however, that a lower level of empowerment can be a marker of social disengagement when the ability of older persons to mobilize needed resources is limited.

The last objective of this paper was to assess the prevalence of unmet needs at the end of the study. Overall, irrespective of area, we found a high prevalence of unmet needs. In the ISD area, unmet needs decreased, dropping from 68% to 35% over time. In the control area, the percentage of participants with unmet need was never lower than 56% and reached 67% at the end of the study. These values, however, are fairly equivalent to estimates reported in recent studies with similar populations [13,35]. Not surprisingly, considering the study population, all five domains covered by the SMAF were compromised. Like numerous studies, we found many unmet needs in the ADL and mobility domains. Inadequate public or private services may make it harder for informal caregivers to satisfy all needs, especially for the more intimate daily activities such as bathing, grooming, toileting, and continence. We also observed several unmet needs in communication functions. At the end of the study, our percentages were, respectively, 11% and 45% for those in the ISD area and those in the control area. It should be mentioned that these activities require constant help throughout the day that even close relatives cannot always provide for. The higher level of unmet needs in the control area may reflect some accessibility problems with medical or rehabilitation services in an area that did not have a coordinated model of services. Moreover, a study investigating the capacity of providers to offer accessible health care for people with disabilities revealed that people with communication limitations or visual impairment were the most difficult to serve [58]. Until now, only one study has investigated unmet needs related to communication functions in a community-dwelling sample of people with dementia, revealing 9% of unmet needs [57]. Lastly, our study found fewer unmet needs than what was reported in prior studies about IADLs, specifically those that could be performed weekly or monthly, such as cleaning the house. These activities are possibly easier to fulfill than more frequent activities such as preparing meals or taking medication.

It is difficult to compare estimates across studies because of differences in study purpose and methods, such as sample characteristics, definitions of disability and unmet needs, type of activity considered, and data-collection approach (e.g. expert evaluation or self-reports). Our relatively high rates of unmet needs, however, may be explained by many factors. First, participants recruited in this study were identified at risk of functional decline, and people with cognitive impairments were not excluded. Second, the SMAF covers a broader spectrum of activities than traditional ADL/IADL domains and includes communication, mobility and mental functions, thereby increasing the possibility of finding unmet needs. Third, we defined unmet needs as both the absence of and the insufficiency of assistance with functional needs. Lastly, unmet needs were assessed by expert clinicians and not self-rated. As underlined by Sands et al [19], in this way, an unmet need is considered a normative need, and estimates are different from what can be found in other studies in which unmet needs were often determined through participant self-reporting [9,10,14,16,17,21]. For example, Morrow-Howell, Proctor & Rozario [59] highlighted the fact that older persons rate their care as more sufficient than professionals do. This situation was also observed with elderly with cognitive problems for whom caregivers were unwilling to report unmet needs, fearing that doing so would negatively reflect on their own caregiving adequacy [10]. Thus, professionals assess needs and unmet needs differently than individuals, but we don't know if either is necessarily better than the other, since they have their own different perspectives. Nevertheless, this factor and all the others taken together may explain why our estimates of unmet needs were higher than estimates from previous studies.

While we attempted to conduct a thorough analysis of the available data, our study was also subject to some limitations. The vast majority of participants were Canadian-born and French-speaking, and this lack of ethnic heterogeneity limits the generalizability of our findings. Many studies [16,17,21,23] have related minority membership to unmet needs. Neither did we investigate economic status, which could be another limitation. Evidence also exists that unmet needs may be associated with the economic status of patients [9,10]. Although Canada has a universal publicly funded health-care system, a study conducted in the province of Quebec using standardized assessments and data from administrative databases revealed that home-care users received only 8% of required services from the public sector [60]. In our study, the rate of services supplied by the public sector was also very low. As supported by Komisar, Feder and Kasper [13], older adults in lower-income households may not be able to supplement publicly funded services with privately funded ones in the face of inadequate informal help.

In counterpart, many of this study's strengths bolster our confidence in these findings. They include the use of a representative community-based sample, a longitudinal design in which participants were interviewed annually by trained research interviewers with face-to-face assessments and well validated instruments. In the province of Quebec, the SMAF has been included since 2001 in the Multiclientele Assessment Tool, which has been approved by the government for use in all long-term-care facilities, including home health-care agencies [61]. As the SMAF evaluates the functional ability according to many sources of information, clinical observations have offered additional insight into whether care needs were met during the course of this study. Another advantage of the SMAF is the possibility of simultaneously identifying needs that are met or unmet. Both indicators are useful for the planning and organization of services. Needs assessment examine the type and amount of required services, while assessment of unmet needs highlights problems that are not being addressed by home-care services and their partners. At the clinical level, this information facilitates prioritization of services to meet these needs. For management purposes, such information about unmet needs is useful for monitoring the responsiveness of the service-delivery system. Over the years, many services now provided in Quebec home-care programs became entrenched without having to demonstrate their efficacy in responding to needs related to disabilities.

Loss to follow-up is usually a threat to longitudinal data analysis, since it leads to reduction in sample sizes and to biases. The use of growth modeling, however, enabled us to use all available data for participants lost to death or institutionalization, those with missing data at one or more time points, and to consider data from participants added in 2003. The longitudinal approach has allowed us to observe the dynamics of unmet needs in older persons. Capturing these changes revealed that unmet needs were not necessarily temporary. In specific contexts, some unmet needs are perhaps inevitable, but the high level of unmet needs and the fact that they were not transitory raise some policy issues. ISD networks such as the PRISMA Model, however, seem to be effective in reducing unmet needs, and a study realized in the United States revealed that although unmet needs were substantial in the 6 states surveyed, the broader the access to publicly supported care in a state, the lower the incidence of unmet needs [13].

## Conclusions

In spite of the full range of health and social services that home-care services can provide in the province of Quebec, disabled older adults living in the community continue to express unmet needs in a significant way. Prior studies have determined that living with unmet needs was associated with falls and an increased use of emergency and acute health-care services [10,14]. Sands et al. [19] has shown that meeting disabled older peoples' unmet needs can resolve these health consequences. Therefore, monitoring elders according to their needs and minimizing unmet needs should be primary goals underlying our long-term care policy. Although it is challenging to meet elders' home care needs completely, strategies need to be developed and implemented to better address them. Regardless of who provides home-care services, the assistance level seems to be important. In this way, ISD networks corresponding to the PRISMA Model appear to be effective ways of meeting the long-term care needs of older adults, especially people with higher levels of disability. Thus, we must urgently adapt our operations and resources through the use of case management, a single entry point, a unique assessment tool, and an individualized service plan to promote better coordination between services. This also highlights the role of case managers, who have to explore all possible sources for help and to find the adequate amount of services, while facilitating access to them.

## Competing interests

The authors declare that they have no competing interests.

## Authors' contributions

Author ND contributed to the study's conception and design, conducted the literature review, oversaw the statistical analyses, interpreted the data, and had primary responsibility in drafting the manuscript. MFD contributed to the study's conception and design, participated in drafting the manuscript, and made substantial contributions to the supervision of the statistical analyses and data interpretation. MR coordinated the practical training of interviewers and data collection, and helped with interpret the data. NRG performed the statistical analyses and helped interpret the data. RH conceived, directed, and supervised all steps of the original study and helped interpret the data. All authors critically revised the manuscript, and read and approved the final manuscript.

## Pre-publication history

The pre-publication history for this paper can be accessed here:

http://www.biomedcentral.com/1471-2318/11/67/prepub
